# Development of a Simple Reversible-Flow Method for Preparation of Micron-Size Chitosan-Cu(II) Catalyst Particles and Their Testing of Activity

**DOI:** 10.3390/molecules25081798

**Published:** 2020-04-14

**Authors:** Apichai Intanin, Prawpan Inpota, Threeraphat Chutimasakul, Jonggol Tantirungrotechai, Prapin Wilairat, Rattikan Chantiwas

**Affiliations:** 1 Department of Chemistry and Center of Excellence for Innovation in Chemistry, Faculty of Science, Mahidol University, Rama VI Rd., Bangkok 10400, Thailand; apichai.itn@gmail.com (A.I.); praw.inpota@gmail.com (P.I.); threeraphat.chu@gmail.com (T.C.); jonggol.jar@mahidol.ac.th (J.T.); 2 National Doping Control Centre, Mahidol University, Rama VI Rd., Bangkok 10400, Thailand; prapin.wil@mahidol.ac.th

**Keywords:** flow method, chitosan, catalyst particles, micron-size, sampling study, *p*-nitrophenol reduction

## Abstract

A simple flow system employing a reversible-flow syringe pump was employed to synthesize uniform micron-size particles of chitosan-Cu(II) (CS-Cu(II)) catalyst. A solution of chitosan and Cu(II) salt was drawn into a holding coil via a 3-way switching valve and then slowly pumped to drip into an alkaline solution to form of hydrogel droplets. The droplets were washed and dried to obtain the catalyst particles. Manual addition into the alkaline solution or employment of flow system with a vibrating rod, through which the end of the flow line is inserted, was investigated for comparison. A sampling method was selected to obtain representative samples of the population of the synthesized particles for size measurement using optical microscopy. The mean sizes of the particles were 880 ± 70 µm, 780 ± 20 µm, and 180 ± 30 µm for the manual and flow methods, without and with the vibrating rod, respectively. Performance of the flow methods, in terms of rate of droplet production and particle size distribution, are discussed. Samples of 180 µm size CS-Cu(II) particles were tested for catalytic reduction of 0.5 mM *p*-nitrophenol to *p*-aminophenol by 100-fold excess borohydride. The conversion was 98% after 20 min, whereas without the catalyst there was only 14% conversion.

## 1. Introduction 

Heterogeneous metal catalyst has been widely used in polymer and chemical production and in petroleum, pharmaceutical, and food industries [1,2]. They provide advantages such as ease of removal and recovery for recycling [3]. Recently, methods have been developed for preparation of heterogeneous catalyst particles with different sizes (ranging from nanometers to millimeters) and various shapes (spherical, rod, and fiber) [4,5,6,7]. Chitosan (CS) is a natural biopolymer. It can be obtained from alkaline deacetylation of chitin contained in the exoskeletons of crabs, shrimps, or insects. Chitosan is composed of polysaccharides and heteropolymers of glucosamine and acetylglucosamine, with pKa values in the range of 6.3–6.7 [4]. Chitosan has many applications because it is hydrophilic, non-toxic, biodegradable, eco-friendly, and cost-effective. It is thermally stable and can be chemically modified. It also has many metal-binding functional groups with a high affinity for various metal ions, such as Pb(II) [8], Ru(III) [8], Cu(II) [9], and Au(III) [10,11,12]. 

The preparation of catalyst particles can be carried out to give a range of sizes (millimeter, micrometer, and nanometer sizes [13]) for different applications [14]. In general, nano-size catalyst particles provide a greater catalytic activity for the same mass of catalyst when compared to micron-size or milli-size particles. However, reusing nanometer catalysts requires more procedure since it needs either evaporation using solvent or ultrafiltration. Hence, its reusability is a major issue especially for industrial applications in terms of cost-effectiveness or environmental impact. The millimeter- or micrometer-size catalysts can be separated simply by gravity settling for reuse. The micron- to milli-size particles are prepared by different methods, such as spray drying [15], jet cutting [14,16], and manual addition [17,18].

Manual dropping method is the conventional method for the preparation of millimeter-size particles [17,18]. The procedure produces hydrogel drop by drop. The limitations of manual dropping, spray drying, and jet cutting method are difficulty in scaling-up, low production rate, and difficulty in controlling size and shape, especially with the manual dropping method [17,18,19,20,21,22]. The methods (i.e., spraying, jet cutting) can be carried out in continuous flow procedure. Automation control is possible for spraying method, although it is complicated to set up [23]. Jet cutting method is suitable for a wide range of viscous fluidic medium and is designed for industrial scale production of monodisperse beads for pharmaceutical or biotechnology applications [14]. 

Micron-size particles exhibit more catalytic activity than millimeter-size catalysts. Simple preparation method can be used, such as manual dropping method, to produce these particles. A further advantage of using micron-size particle is the convenience of reusability without the need for separation via settling or filtration. Thus, this work aims to produce uniform micron-size CS-Cu(II) catalyst particles with narrow size distribution employing a simple flow method. It is efficient to produce the uniform with consistent size of synthesized catalyst due to the designs of flow instruments are promising with various platforms for flow synthesis [24]. Chitosan is employed as the substrate for synthesizing the heterogeneous metal catalysts. Copper is one of the most widely used transition metals for preparation of either homogenous or heterogeneous catalyst [25]. It has high activity with a wide range of industrial applications [26]. A simple reversible-flow system incorporating a vibrating rod was developed and compared with the flow method without the vibrator and also with the manual dropping method. A sampling procedure was employed to provide representative samples of the CS-Cu(II) particles for size analysis using images recorded under an optical microscope and analyzed with ImageJ program. The synthesized CS-Cu(II) catalyst particles were evaluated for their catalytic activity by using the well-known reaction of reduction of *p*-nitrophenol by aqueous borohydride solution to give the aminophenol product. *p*-nitrophenol has been used as a chemical in various industrial manufacturing processes, such as analgesic and antipyretic drugs, photographic developer, corrosion inhibitor, and dyes. However, it is hazardous and causes serious hypoxia. It has major effects on blood, liver, and the central nervous system [27]. Because of the extensive use of this compound, it is selected as the model compound for testing the catalytic property of the synthesized material. This development of a simple and convenient set-up of a flow method for preparation of heterogeneous catalyst of reproducible size on a small scale is convenient and efficient. It is less time consuming and more cost-effective for synthesis of various possible hydrogel formulations. This would lead to faster testing of the activity of the catalysts with different chemical reactions.

## 2. Results and Discussion

### 2.1. Evaluation of Sampling Procedure for Measurement of Particle Size of CS-Cu(II) Catalyst Particles 

**Figure 1:**
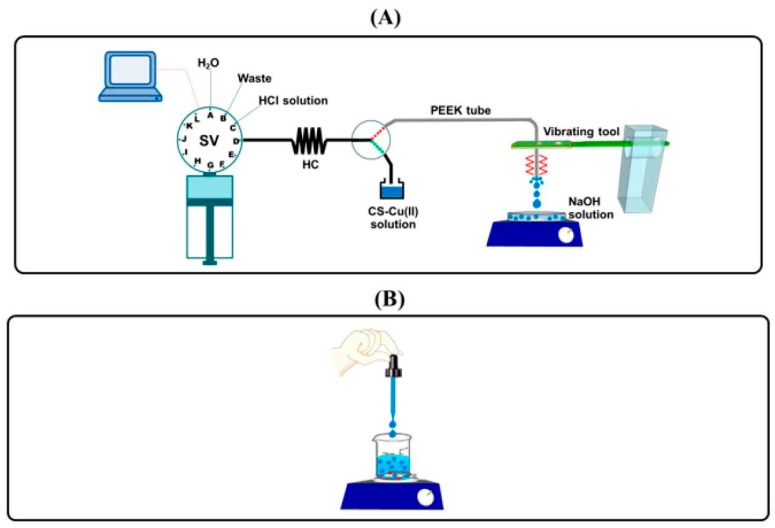
(**A**) Schematic of the flow system for synthesis of CS-Cu(II) catalyst particles with the PEEK tubing inserted through the vibrating rod. The reagent droplets at the tip of the vibrating tubing falls into a solution of NaOH. SV: selection valve, HC: holding coil. (**B**) Manual dropping method using a Pasteur pipette with a rubber teat.

**Figure 2:**
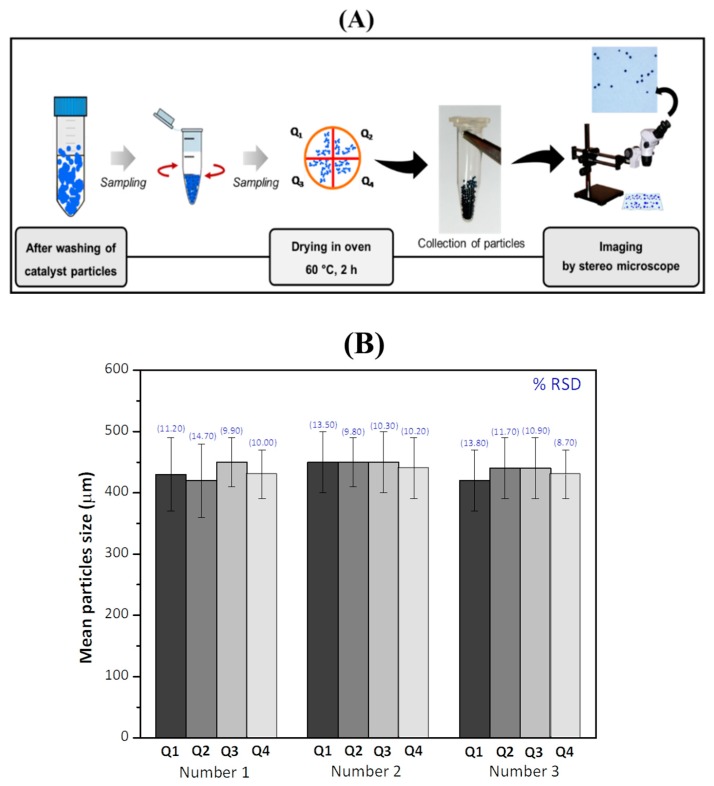
(**A**) Schematic of the sampling procedure for particle size measurements of the CS-Cu(II) particles. Bulk catalyst particles (~10.8% *w*/*v*) are vortexed before pipetting 0.3 mL of suspension into a microtube. Then, 0.3 mL of suspension is pipetted onto a filter paper, dried and divided into 4 quarters before drying in an oven at 60 °C (2 h). Particles in each quarter collected for recording of image on an optical microscope and size measurement using ImageJ software. (**B**) Bar graphs of mean particle size and % RSD for each quarter (Q1–Q4) and 3 sampling aliquots (Number 1–Number 3).

The study of sampling procedure was carried out by employing the reversible flow system for synthesis of CS-Cu(II) catalyst particles, see Figure 1A. The sampling procedure (see Section 3.3 and Figure 2A) provided particles for determination of the size distribution using ImageJ software analysis of the recorded images taken on an optical stereo microscope. As shown in Figure 2B, the mean particle size of 30 particles in each sector (Q1–Q4) for all 3 aliquots (Number 1–3) is in the range of 390–490 μm. The average of the mean particle size of the sectors (*n* = 12) is 440 ± 50 µm. Thus, the sampling procedure is effective for obtaining representative particles for particle size measurements.

### 2.2. Investigation of Flow Parameters

Figure 1A shows the schematic of the flow system with the vibrating tool for the preparation of the CS-Cu(II) catalyst particles, see Table 1 for details of operational flow procedure. Various flow parameters can affect the particle shape and size distribution, i.e., the chitosan content, the NaOH concentration, and the flow rate of the CS-Cu(II) reactant solution. These parameters were studied with the objective of obtaining uniform size and shape of the catalyst particles.

#### 2.2.1. Effect of Chitosan Content

**Figure 3:**
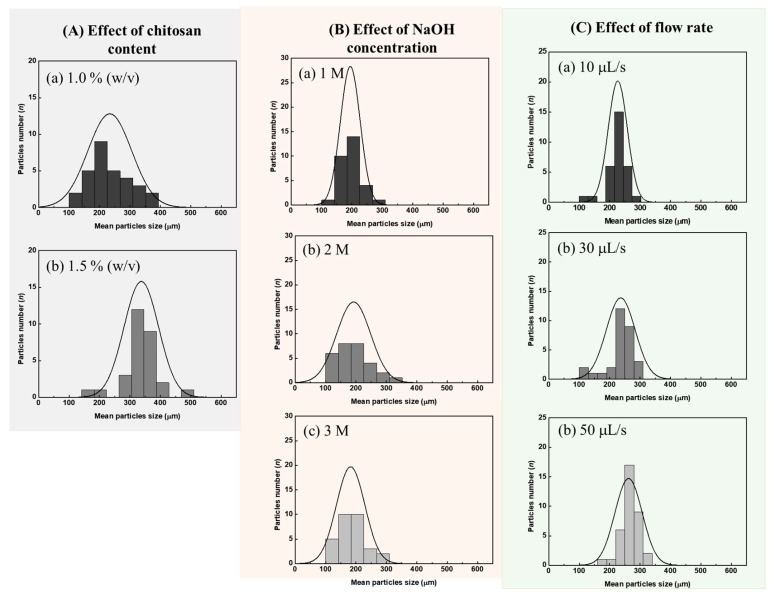
Histograms of the particle size distribution of CS-Cu(II) catalyst particles produced under various flow conditions. (**A**) Effect of chitosan content: 1.0 1.5% (*w*/*v*). Chitosan at 0.5% and 2.0% did not produce hydrogel droplets due to the low and high viscosity of the reagent solutions, respectively. (**B**) Effect of NaOH concentration: (a) 1 M, (b) 2 M, and (c) 3 M NaOH is used for solidification of chitosan-Cu(II) catalyst particles. (**C**) Effect of flow rate of the CS-Cu(II) reagent: (a) 10, (b) 20, and (c) 30 μL s^−1^, respectively.

The chitosan concentrations were varied from 0.5% to 2.0% (*w*/*v*) (see Section 3.1 for preparation of solution). Catalyst particles were formed with increasing size (230 ± 70 µm to 340 ± 40 µm) when the chitosan concentration was increased from 1.0% to 1.5% (*w*/*v*) (see Figure 3A). However, at 0.5% (*w*/*v*), chitosan did not form hydrogel droplets because of its low viscosity. Conversely, the high viscosity of the 2.0% (*w*/*v*) solution limited the flow of the chitosan solution. Hence, histograms of the size distribution for the 0.5% and 2.0% (*w*/*v*) chitosan solutions were not constructed. The concentration of chitosan solution of 1.5% (*w*/*v*) was selected since it gave particles with more spherical shape and narrower size distribution than the 1.0% (*w*/*v*) chitosan sample (see Figure 3A).

#### 2.2.2. Effect of NaOH Concentration

Suitable NaOH concentration was investigated since the concentration of NaOH of the receptacle solution (Figure 1A) affects the rate of chitosan solidification. An optimum solidification rate is needed to obtain a narrow particle size distribution. Chitosan is soluble in hydrochloric acid solution due to the protonation of amino groups in the polymeric chain. In alkaline solution, the protons on the polymeric chain are dissociated and the neutral polymer chain can aggregate together. Concentrations of the NaOH solutions selected were 1 M, 2 M, and 3 M, respectively. Figure 3B shows the results obtained for the different NaOH concentrations. The use of 1 M and 2 M NaOH solutions gave similar results for the mean size of the particles, viz. 260 ± 50 µm and 240 ± 50 µm, respectively. Particle size distributions were narrower for the 1 M and 2 M NaOH solutions than for the 3 M solution. Therefore, 2 M NaOH was selected as the operating NaOH concentration.

#### 2.2.3. Effect of Flow Rate of the CS-Cu(II) Reactant Solution

The flow rate of the CS-Cu(II) solution was varied from 10 µL s^−1^ to 50 µL s^−1^. The flow rate of 10 µL s^−1^ is the smallest rate possible for the syringe pump. The resulting size distribution of the synthesized CS-Cu(II) catalyst particles are shown in Figure 3C. The catalyst particles have mean sizes of 230 ± 30 µm, 240 ± 50 µm, and 260 ± 40 µm, when the flow rates are 10 µL s^−1^, 30 µL s^−1^, and 50 µL s^−1^, respectively. Therefore, the mean size of the CS-Cu(II) catalyst particles is not significantly different with increasing flow rate. On the other hand, the size distribution increases with the flow rate. Therefore, the flow rate for the reagent solution of 10 µL s^−1^ was selected.

The parameters of the flow system studied above are summarized in Table 2. The selected conditions are given in the last column of Table 2.

### 2.3. Comparison of Methods for Synthesizing CS-Cu(II) Catalyst Particles: Flow Methods vs. Manual Addition

**Figure 4:**
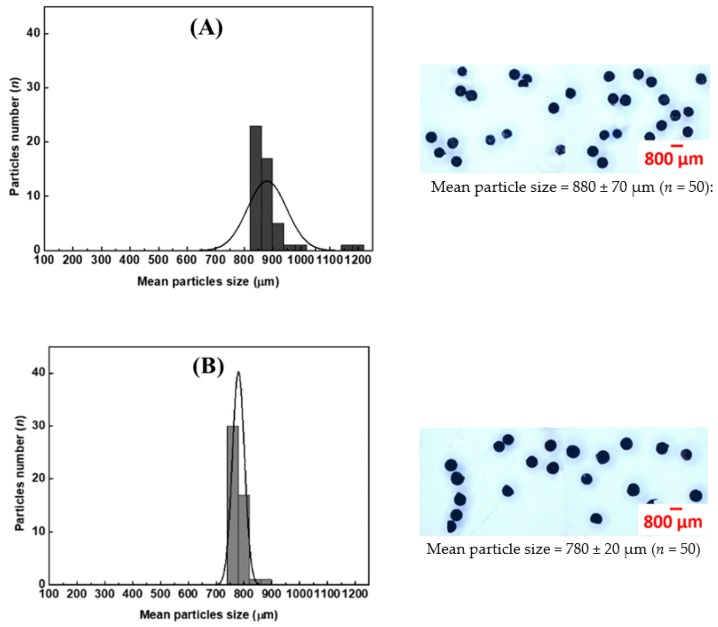

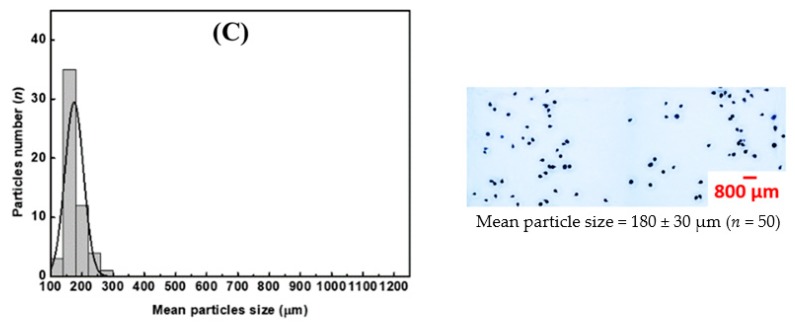
Particle size distributions with images of representative particles obtained using different flow methods. (**A**) Manual dropping method, (**B**) flow method without vibration and (**C**) flow method with the vibrating rod. The scale bar for 800 μm length is shown.

Three methods for the preparation of the CS-metal catalyst particles were investigated, i.e., flow method with a vibrating rod (see Figure 1A), flow without the vibrating rod, and the manual dropping method (see Figure 1B). The resulting products were collected, dried, and sampled, and the size distribution measured as described in Section 3.3. Figure 4 shows the distributions of the particle size of the CS-Cu(II) catalyst and images of the particles as prepared by the three methods. A much smaller particle size (150–210 µm) was obtained by the flow method using the vibrating rod. There was greater uniformity of particle shape and narrower size distribution. The vibrating rod assisted in producing smaller droplets with a 4-fold reduction in the particle size than that obtained without vibration (780 ± 20 µm vs. 180 ± 30 µm), see Figure 4B,C.

The manual dropping method is the conventional method for synthesis of the particles. The mean particle size was 880 ± 70 µm (see Figure 4A). Drawbacks of this method are the difficulty in controlling the resulting size and shape of particles and the low production rate. The size and number of drops depend on the manual pressure applied to the rubber bulb. The throughput of this method is about 40 drops min^−1^. 

The flow-based method provides advantages such as precision of flow rate and automation of operation. Therefore, the flow method with the vibrating rod was employed for producing micron-size uniformly shaped particles of CS-Cu(II) catalyst particles. 

Comparison of the performance between manual dropping method and flow methods, with and without vibrating rod, is shown in Table 3.

### 2.4. Characterization of CS-Cu(II) Catalyst Particles

A SEM picture of the micron-size CS-Cu(II) catalyst is shown in Figure S1a, Supplementary Material A [28]. The surface morphology of the particle shows unique streaking patterns with uniform surface structure. Figure S1b and S1c show characteristic FTIR spectrum and XRD pattern of the catalyst, respectively. The FTIR spectrum has bands at 3430, 2868, 2364, 1622, 1379, and 1097, 1013, and 599 cm^−1^. These are the “fingerprint” of chitosan and the strong broad peak at 3430 cm^−1^ is the hydrogen-bonded O-H stretching band. The XRD pattern reveals low-intensity peaks at 2θ = 35.4° and 38.8°, designated to (002) and (111) reflections of CuO, respectively. Nitrogen adsorption/desorption isotherms, as shown in Figure S1d, indicate non-porous structure with low surface area.

### 2.5. Assessment of the Catalytic Activity of the Chitosan-Cu(II) Particles in the Reduction Reaction of p-Nitrophenol with Excess Borohydride

**Figure 5:**
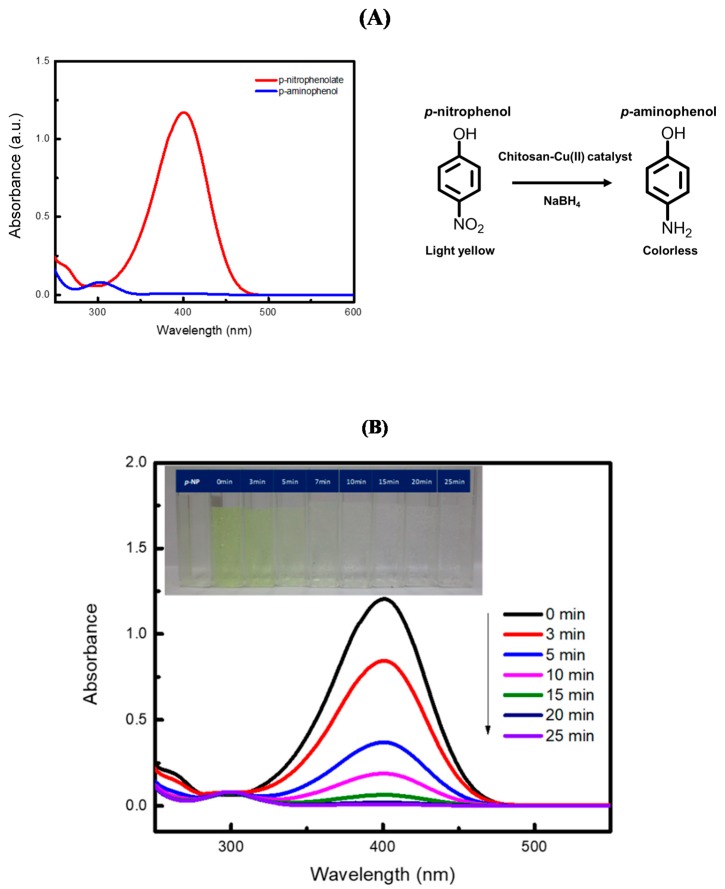
(**A**) Spectra of the reduction reaction of *p*-nitrophenol with 100-fold excess sodium borohydride and 0.2 mg/mL CS-Cu(II) catalyst particles (180 μm mean size). The red spectrum is the initial spectrum of the *p*-nitrophenolate anion (83 μM) produced rapidly on addition of borohydride. The blue spectrum is the spectrum at the end of the reaction. (**B**) UV–Visible absorption spectra and photographs (inset) of the filtered and diluted (5-fold) reaction solution sampled at various times. The initial concentrations are 0.5 mM *p*-NP with 0.05 M NaBH_4_ solutions containing 0.2 mg mL^−1^ CS-Cu(II) catalyst particles (180 μm mean size).

The reduction of *p*-nitrophenol (*p*-NP) with excess borohydride is a common chemical reaction employed for testing the catalytic activity of heterogeneous metal catalysts [29]. The net chemical reaction is shown in Figure 5A. When a *p*-nitrophenol solution is mixed with a solution of NaBH_4_, *p*-nitrophenol is first rapidly deprotonated to give the *p*-nitrophenolate anion as an intermediate, which is then reduced to *p*-aminophenol. The UV–visible spectra of *p*-nitrophenolate anion and *p*-aminophenol are shown in Figure 5A. The reaction can be monitored by measurement of absorbance at 400 nm. The rate of the reaction without catalyst particles is slow. Metal catalysts, such as Au [30], Ag [31], and Cu [9], have thus been used to accelerate the reaction.

In this work, CS-Cu(II) particles were assessed as catalyst for the above reduction reaction. Spectra of a solution of *p*-NP containing the catalyst were measured at various time intervals, as shown in Figure 5B. The *p*-nitrophenol solution is pale yellow. On addition of sodium borohydride (NaBH_4_), the color of the solution is changed to bright yellow due to the rapid formation of the *p*-nitrophenolate anion (see the first and second cuvettes from the left in the inset of Figure 5B. There is a red shift of the wavelength at the maximum absorbance from 317 nm to 400 nm (data not shown). The absorbance of *p*-nitrophenolate at 400 nm decreases with time with a small concomitant increase of the absorbance of the *p*-aminophenol product at 300 nm. Percent conversion of *p*-nitrophenol was calculated from $\frac{\left(A_{0}-A_{t}\right)}{A_{0}}\times 100$, where A_0_ is the initial absorbance of *p*-nitrophenolate anion, and A_t_ is the absorbance of *p*-nitrophenolate at different time intervals. Figure 5B shows the photographs (inset) of the solutions of 1.0 mM *p*-nitrophenol with CS-Cu(II) catalyst before addition of borohydride and after addition at 0, 3, 5, 10, 15, 20, and 25 min, respectively. The measured UV–visible spectra at 0–25 min are also shown. At 25 min, there was 98% conversion (t_1/2_ ca. 4 min) as compared with less than 14% conversion (t_1/2_ ca. 115 min) without the catalyst (data not shown). Our conversion efficiency (>90%) was comparable with previous reports using the same reaction of *p*-nitrophenol catalyzed by other synthesized catalysts, such as activated carbon-supported gold nanocatalysts (Au/AC) [32] and magnetic chitosan-supported silver nanoparticles [33]. Further detailed kinetics study, together with employment of other compounds, are part of future studies.

## 3. Materials and Methods

### 3.1. Chemicals and Preparation of Chitosan and Cu(II) Solutions

All chemicals used in the experiments were of analytical grade (AR). Chitosan powder, copper(II) acetate monohydrate (98% assay), and sodium hydroxide were from Sigma-Aldrich (St. Louis, MO, USA). Dimethyl sulfoxide (99.9% assay) and hydrochloric acid (37% assay) were obtained from RCI Labscan (Bangkok, Thailand). *p*-Nitrophenol (99.5% assay) was purchased from Merck (Darmstadt, Germany). Sodium borohydride (98% assay) was from Alfa Aesar (Tewksbury, MA, USA). Ultrapure water was from a Milli-Q^®^ Advantage A10 water purification system (Darmstadt, Germany).

The chitosan and copper acetate solutions were prepared freshly. Chitosan solution (1.5% *w*/*v*) was prepared by dissolving chitosan powder in 10.00 mL of 0.2 M HCl with continuous stirring for 30 min. Then, 4.0 mL of the Cu(II) solution (2.0 M) was added dropwise to the chitosan solution while stirring continuously for 45 min. Then, 0.5 mL DMSO was added into the CS-Cu(II) solution, which was then stirred for 15 min and sonicated for an hour.

### 3.2. Preparation of CS-Cu(II) Catalyst Particles

#### 3.2.1. Configuration and Operation of the Flow System 

Figure 1A shows the manifold of the flow system with the vibrating tool for the preparation of micron-size CS-Cu(II) catalyst particles. The flow system comprises a syringe pump with a 5.0 mL syringe barrel (Norgren Kloehn Inc., Las Vegas, NV, USA). A 3-way switching valve is used to draw the sample into the holding coil (HC) (FEP tubing, 2.0 mm i.d., 159 cm long). The flow line connecting the SV and the HC is a PTFE tubing (0.5 mm i.d., 26 cm long). The 3-way switching valve is also connected to a PEEK (polyether ether ketone) tubing (0.75 mm. i.d., 48 cm long), which passes through a hole at the paddle end of the vibrating rod. The length of the PEEK tubing extending from the vibrating rod is set at 5.0 cm. The tip of the PEEK tubing is placed above the container of the NaOH solution (see Figure 1A). 

The vibrator is a commercial electric toothbrush (Systema Sonic, Lion, Japan). The step-by-step operation is set through the software controller and is listed in Table 1. A 4000 μL solution of CS-Cu(II) reagent is pumped slowly (10 μL s^−1^) to form a small drop at the tip of the PEEK tubing. The drop then breaks off into the NaOH solution, leading to the formation of hydrogel droplets. The resulting hydrogels were washed and dried to obtain CS-Cu(II) catalyst particles. The dry product was analyzed for particle size distribution as described in Section 3.3 (see Supplementary Material B (Figure S2) and Table S1 for detail of ImageJ software).

#### 3.2.2. Manual Dropping Method

Schematic of the manual dropping method for preparation of the particles is shown in Figure 1B. The CS-Cu(II) solution was added to the NaOH solution dropwise with a Pasteur pipette using a rubber teat. The hydrogel particles were then filtered and washed with deionized water prior to analysis for shape and size distribution (see Section 3.3).

### 3.3. Sampling and Measurement of Particle Size of Synthesized CS-Cu(II) Catalyst 

The freshly synthesized CS-Cu(II) particles were added to deionized water to give a concentration of 10.8% (*w*/*v*) and vortexed. Then, three 0.3 mL aliquots of the suspension were pipetted and transferred into 3 microtubes (1.5 mL) and 0.70 mL ultrapure water added to each tube. After vortexing, 0.3 mL of the suspension from each tube was pipetted onto 3 separate filter paper (Whatman no. 2) to absorb the water and then dried in an oven at 60 °C for 2 h (see Figure 2A). The dry particles were divided into 4 sections, and particles in each section collected in individual vials. Images of particles from each vial were recorded on a stereomicroscope at 2× magnification (OLYMPUS SZ51, Tokyo, Japan). The digital image was analyzed by ImageJ software to obtain the mean size of the particles. Threshold number of the ImageJ software was adjusted ± 30 from the default threshold number (see details in Supplementary Material B).

### 3.4. Method of Characterization of CS-Cu(II) Catalyst Particles 

Characterization of CS-Cu(II) catalyst was carried out using various techniques, e.g., scanning electron microscopy (SEM) using a Hitachi S-2500 instrument, Fourier-transform spectroscopy (FTIR) employing KBr disc with a PerkinElmer spectrometer, x-ray diffraction (XRD) using a Bruker D8 Advance diffractometer, and nitrogen adsorption/desorption isotherms and pore size distribution using Quantachrome AUTOSORB-1 system. The results are shown in Supplementary Material A [28].

### 3.5. Procedure for Measuring Activity of CS-Cu(II) Particles Using Reduction Reaction of p-Nitrophenol 

A solution of 1.0 mM *p*-nitrophenol was prepared by dissolving 0.0696 g of *p*-nitrophenol powder in ultrapure water and then adjusted to volume in a 500.0 mL volumetric flask. Sodium borohydride (0.1 M NaBH_4_) was used as the reducing agent by dissolving 0.1892 g NaBH_4_ in 50.0 mL of DI water. A 25.0 mL volume of 1 mM *p*-nitrophenol solution was mixed with 25.0 mL of 0.1 M NaBH_4_ solution and continuously stirred for 30 s. Then, 10.0 mg of the dry 180 μm-size CS-Cu(II) particles were added to the reaction solution with continuous stirring and the timing of the reaction started. At various time intervals (0, 3, 5, 10, 15, 20, and 25 min), 1.5 mL aliquot of the reaction solution was removed and filtered with a syringe filter. Then, 1.00-mL of the filtrate was mixed with 5.00 mL of ultrapure water and the absorbance measured on a UV–visible spectrophotometer at 400 nm. The reaction solution was observed to change from bright yellow to colorless over time (see inset Figure 5B). The UV–visible spectra of the solutions at various time intervals are shown in Figure 5B.

## 4. Conclusions

This work presents the development of a simple flow method for the synthesis of uniform micron-size CS-Cu(II) catalyst particles, together with testing of their catalytic activity for the reduction reaction of *p*-nitrophenol with excess borohydride. The flow system comprises a reversible-flow syringe pump together with a 3-port switching valve and a holding coil. Various flow parameters, *viz*. chitosan concentration, NaOH concentration, and flow rate, were investigated to produce the smallest micron-size with narrow size distribution of the dry synthesized material. The selected conditions were: 1.5% (*w*/*v*) chitosan, 2.0 M copper acetate, and 2 M NaOH. The most suitable flow rate for adding the reagent solution into the sodium hydroxide was 10 µL s^−1^. The performance of the flow method employing the vibrating rod was compared to that without the vibrator and also to a manual dropping method.

A sampling method of the synthesized particles was employed to obtain representative samples for size measurement. Images of the samples were recorded on an optical microscope and the size and shape of the particles analyzed using ImageJ software. The mean sizes of the catalyst particles were 180 ± 30 µm, 780 ± 20 µm, and 880 ± 70 µm, produced using the flow system with and without the vibrating tool and the manual dropping method, respectively. The flow method with the sonic tool provided narrower size distribution of the particles (150–210 μm) and uniform quasi-spherical particles. The flow methods have higher throughput for preparation of the catalyst particles, with rates of 100 drops min^-1^ using the sonic tool and 60 drops min^−1^ without, respectively. The 180 μm-size CS-Cu(II) particles, at concentration of 0.2 mg mL^−1^, were found to catalyze the reduction of *p*-nitrophenol (*p*-NP) with excess borohydride by more than 30-fold compared to reduction without the catalyst.

## Figures and Tables

**Table 1:** Operation procedure of the flow system shown in Figure 1A for preparation of micron-size chitosan (CS)-Cu(II) catalyst particles.

Step	Port	Flow Rate (µL s^−1^)	Operation (Volume/µL)	Description
Aspirate H_2_O carrier into syringe pump (repeat steps 1–2 twice)				
1	1	50	Aspirate (5000 µL)	Syringe pump valve in; aspirate H_2_O.
2	4	50	Dispense (5000 µL)	Syringe pump valve out; dispense H_2_O.
Aspirate air, CS-Cu(II) catalyst solution into holding coil				
3	4	10	Aspirate (200 µL)	Switch 3-port switching valve; dispense air plug.
4	4	10	Aspirate (4000 µL)	Aspirate plug of CS-Cu(II) catalyst solution.
Dispense CS-Cu(II) catalyst solution, air to the tip of tubing				
5	4	10	Dispense (4200 µL)	Switch 3-port switching valve; dispense CS-Cu(II) catalyst solution and air plugs.
Cleaning of holding coil by 0.2 M HCl solution and H_2_O (repeat steps 6–9 twice)				
6	3	50	Aspirate (5000 µL)	Syringe pump valve in; aspirate 0.2 M HCl solution plug.
7	4	50	Empty	Syringe pump valve out; dispense 0.2 M HCl solution plug.
8	1	50	Aspirate (5000 µL)	Syringe pump valve in; aspirate H_2_O plug.
9	1	50	Empty	Syringe pump valve out; dispense H_2_O plug.

**Table 2:** The concentrations of reagents and flow rates for preparation of CS-Cu (II) catalyst particles.

Parameter	Studied Range	Selected Value
1. Concentration of chitosan (% *w*/*v*)	0.5–1.5	1.5
2. Concentration of copper acetate (M)	1–3	2
3. Concentration of NaOH (M)	1–3	2
4. Flow rate (µL s^−1^)	10–50	10

**Table 3:** Comparison of method performance between manual dropping method, flow method without vibration, and flow method with vibrating rod.

Parameter	Manual Method	Flow Method Without Vibration	Flow Method with Vibrating Rod
1. Operation mode	Manual	Computer control	Computer control
2. Mean particles size (*n* = 50)	880 ± 70 µm	780 ± 20 µm	180 ± 30 µm
3. Size distribution	810–950 µm	760–800 µm	150–210 µm
4. Shape	Quasi-spherical	Spherical	Spherical
5. Throughput	40 drops min^−1^	60 drops min^−1^	100 drops min^−1^

## Supplementary Materials

The following are available online at https://www.mdpi.com/1420-3049/25/8/1798/s1, Figure S1: Characterization of chitosan-Cu(II) catalyst particles: (a) SEM image; (b) FTIR spectrum of chitosan-Cu(II) catalyst showing bands at 3430, 2868, 2364, 1622, 1379, and 1097, 1013 and 599 cm^−1^, respectively; (c) XRD pattern; (d) Nitrogen adsorption/desorption isotherms and (e) Pore size distribution. Figure S2: Overview of steps including: (1) Preparation of CS-Cu(II) catalyst particles, (2) recording of particles image by an optical microscope, and (3) measurement of particles for size by Image J software. Table S1: Step-by-step procedure using Image J software for particle size analysis.
