# Supplementary material for: Development of a Simple Reversible-Flow Method for Preparation of Micron-Size Chitosan-Cu(II) Catalyst Particles and Their Testing of Activity
Source: Molecules. 2020 Apr 14;25(8):1798. doi: 10.3390/molecules25081798 (PMC7221602; doi:10.3390/molecules25081798)
Supplement: Supplementary file 1 [file molecules-25-01798-s001.pdf]

# SUPPLEMENTARY MATERIALS

# Development of a Simple Reversible-Flow Method for Preparation of Micron-size Chitosan-Cu(II) Catalyst Particles and Their Testing of Activity

Apichai Intanin,[1] Prawpan Inpota,[1] Threeraphat Chutimasakul,[1] Jonggol Tantirungrotechai,[1] Prapin Wilairat,[2] Rattikan Chantiwas[1*]

[1]*Department of Chemistry and Center of Excellence for Innovation in Chemistry, Faculty of Science, Mahidol University, Rama VI Rd., Bangkok 10400, Thailand*
[2]*National Doping Control Centre, Mahidol University, Rama VI Rd., Bangkok 10400, Thailand*

[*] Corresponding author

rattikan.cha@mahidol.ac.th, rattikan.cha@mahidol.edu

Tel: +66-2-201-5199 Fax: +66-2-354-7151

## Content

- **Supplementary Material A:** Characterization of CS-Cu(II) catalyst particles
- **Supplementary Material B:** Measurement of particle size by ImageJ software: Effect of threshold number

# Supplementary Material A

## Characterization of CS-Cu(II) catalyst particles

Characterization of chitosan-Cu(II) catalyst particles were carried out by: **(a)** scanning electron microscopy (SEM), **(b)** Fourier transform spectroscopy (FTIR), **(c)** x-ray diffraction (XRD), **(d)** specific surface areas (nitrogen adsorption/desorption isotherms) and **(e)** measurement of pore size distribution [1].

**(a) SEM image**

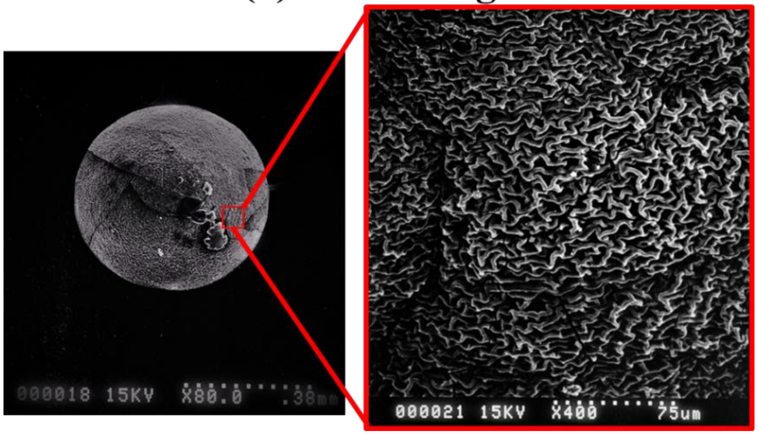


**(b) FTIR spectrum**

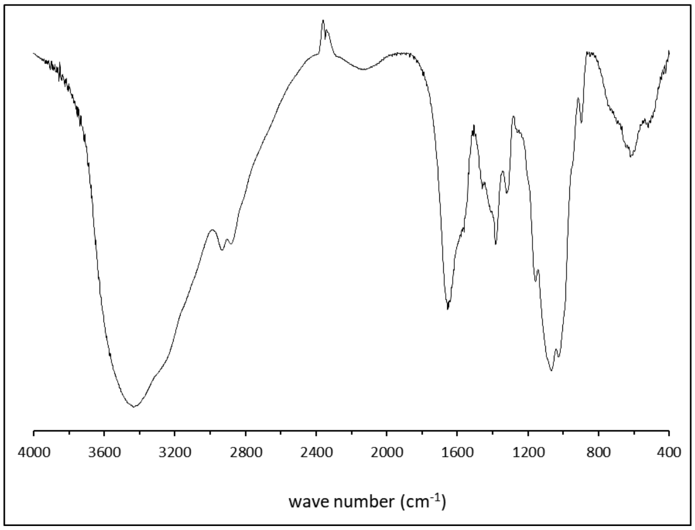

# (c) XRD pattern

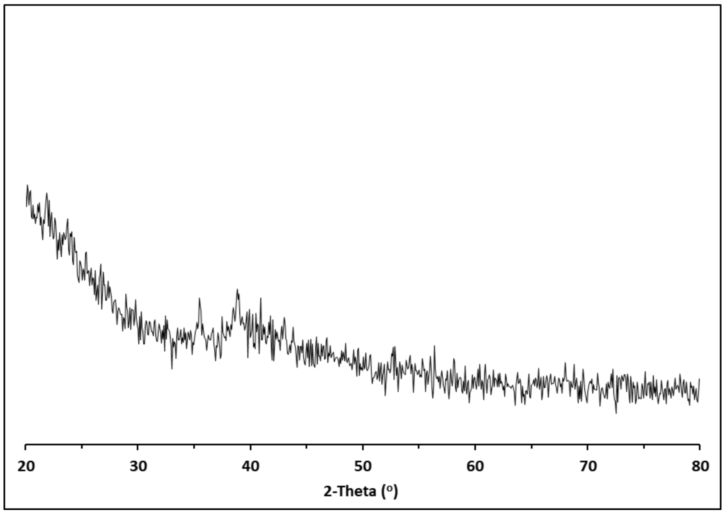


# (d) Nitrogen adsorption/desorption isotherms

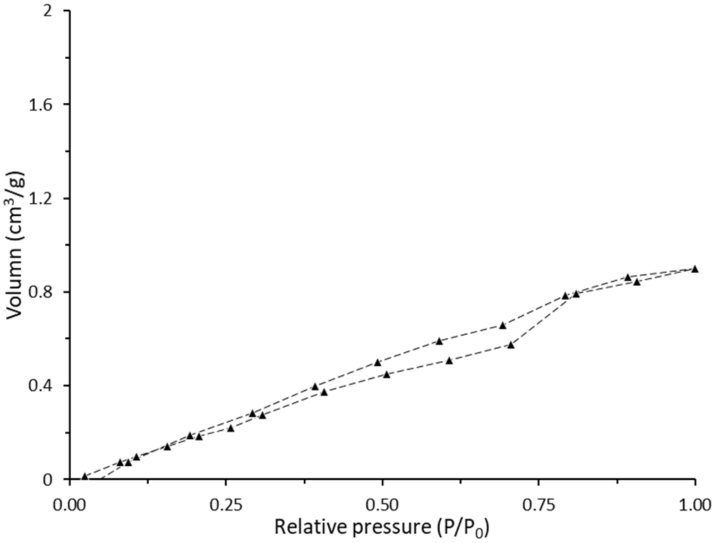

# (e) Pore size distribution

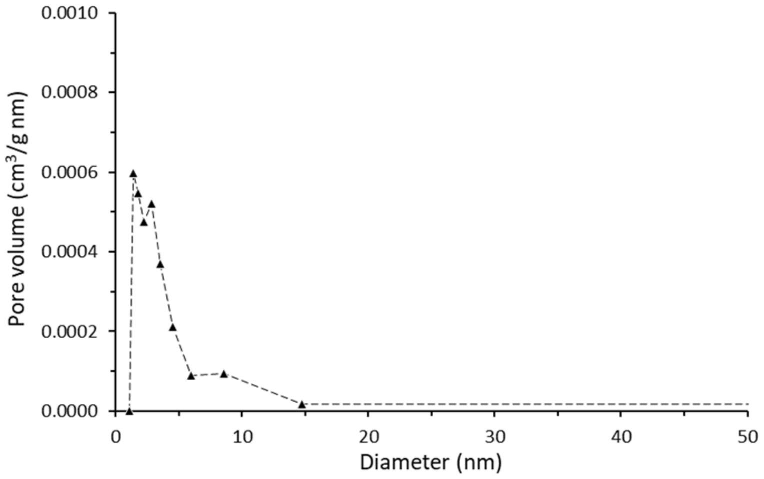


**Figure S1** Characterization of chitosan-Cu(II) catalyst particles: **(a)** SEM image; **(b)** FTIR spectrum of chitosan-Cu(II) catalyst showing bands at 3430, 2868, 2364, 1622, 1379, and 1097, 1013 and 599 $cm^{-1}$, respectively; **(c)** XRD pattern,; **(d)** Nitrogen adsorption/desorption isotherms and **(e)** Pore size distribution.

# Supplementary Material B

## Measurement of particle size by ImageJ software: Effect of threshold number

Image J software is used for analyzing the size of objects on an image and measuring distance between objects. It is a free software for downloading from http://rsb.info.nih.gov/ij/index.html. Image J software is developed by National Institute of Mental Health (NIMH) in United State. It can measure objects in many units, such as square millimeter or number of pixels.

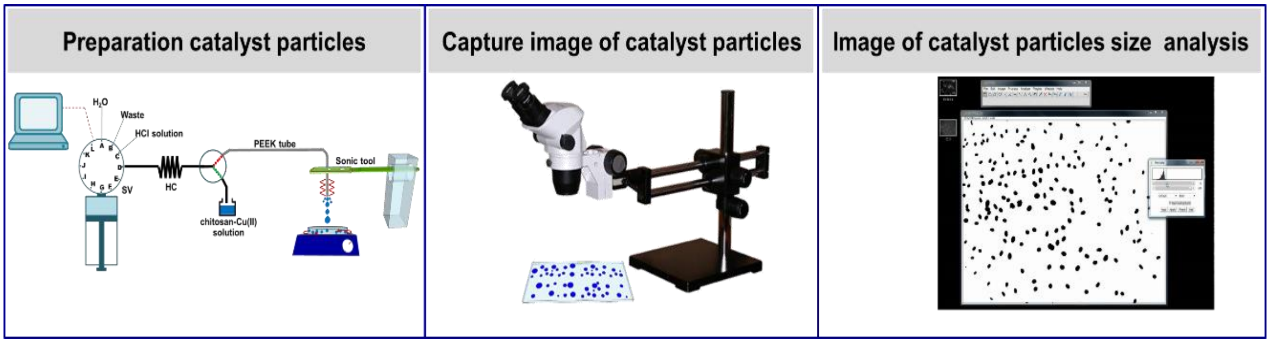


**Figure S2** Overview of steps including: **(1)** Preparation of CS-Cu(II) catalyst particles, **(2)** recording of particles image by an optical microscope, and **(3)** measurement of particles for size by Image J software.

Figure S2 shows the schematic procedure for image analysis. This work uses ImageJ for measurement of particle size of the chitosan-Cu(II) catalyst particles. The operation steps for measurement are shown in Table S1.

Threshold adjustment is a software for segmenting the pixels of an object from the background. Advantages of this software are: 1. reduction of unnecessary pixels of the object, 2. converting image data into bitmap that it is easy to analyze or interpret.

To use the Image J program, the operator has to adjust some parameters before image analysis such as “threshold adjustment”.

**Table S1** Step-by-step procedure using Image J software for particle size analysis.

| Step | Description | Screen capture |
|---|---|---|
| 1 | Opening a digital image file: Go to File ► Open from menu bar to open a digital image of particles file, as shown in picture of the right. | 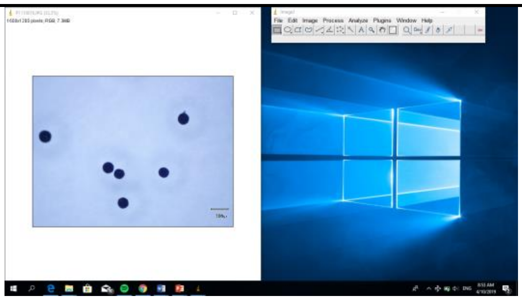 |
| 2 | Setting scale bar in ImageJ: Select a straight line from the menu bar and draw a straight from the known distance on your digital image file, as shown in picture of the right. | 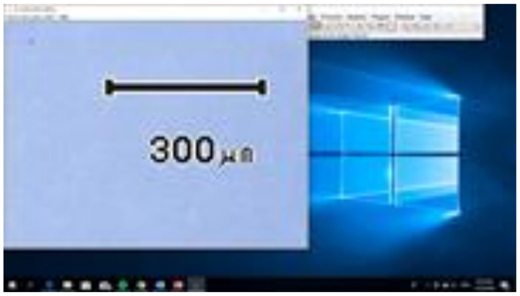  |
| 3 | Converting color digital image (RGB) into grey scale: If your digital image file is color image, it shoud be converted to grey image. Once the image is in grey scale “Image►Type►8-bit”, as shown in in picture of the right. | 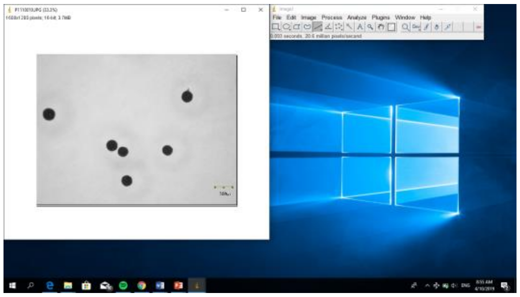 |

| Step | Description | Screen capture |
|---|---|---|
| 4 | Setting a threshold of digital image file: Setting a threshold number to remove the background of image before image analysis (as seen in red color). A threshold number adjustment includes “Image►Adjust ►Threshold, as shown in picture of the right. | 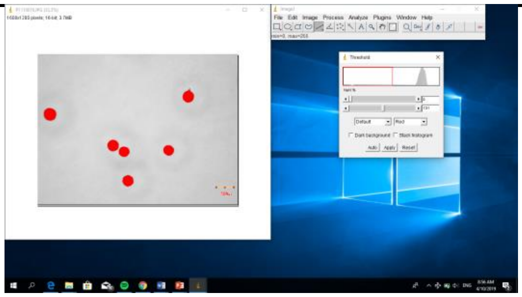 |
| 5 | Measuring area of objects: After finish setting parameter, go to “Analyze ► Analyze Particles”, as shown in Figure A.5. The output of software reports the area. The area data (blue color) was converted to diameter (see picture of the right). | 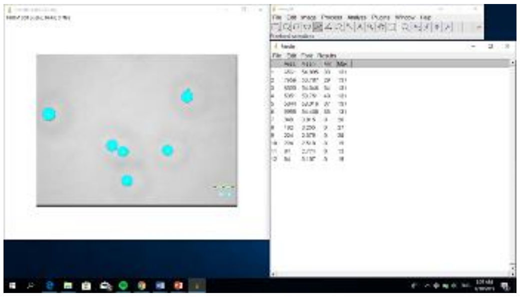 |


**Reference:**

[1]. Chutimasakul, T.; Na Nakhonpanom, P.; Tirdtrakool, W.; Intanin, A.; Bunchuay, T.; Chantiwas, R.; Tantirungrotechai. J. Uniform Cu/chitosan beads as green and reusable catalyst for facile synthesis of imines via oxidative coupling reaction. *Unpublished results*. **2020**.
